# Late Conversion of Kidney Transplant Recipients from Ciclosporin to Tacrolimus Improves Graft Function: Results from a Randomized Controlled Trial

**DOI:** 10.1371/journal.pone.0135674

**Published:** 2015-08-13

**Authors:** Max Plischke, Markus Riegersperger, Daniela Dunkler, Georg Heinze, Željko Kikić, Wolfgang C. Winkelmayer, Gere Sunder-Plassmann

**Affiliations:** 1 Division of Nephrology and Dialysis, Department of Internal Medicine III, Medical University of Vienna, Vienna, Austria; 2 Center for Medical Statistics, Informatics and Intelligent Systems, Medical University of Vienna, Vienna, Austria; 3 Section of Nephrology, Baylor College of Medicine, Houston, Texas, United States of America; Medical University of Graz, AUSTRIA

## Abstract

**Background:**

Tacrolimus (TAC) to ciclosporin A (CSA) conversion studies in stable kidney transplant recipients have reported varying effects on graft function. Here we study graft function (eGFR) trajectories using linear mixed models, which provide effect estimates on both slope and baseline level of GFR and offer increased statistical power.

**Methods:**

Secondary analysis of a randomized controlled trial of CSA treated kidney transplant recipients with stable graft function assigned to receive 0.1 mg/kg/day TAC (target 5–8 ng/ml) or to continue CSA based immunosuppression (target 70–150 ng/ml) at a 2:1 ratio. Renal graft function was estimated via the MDRD (eGFR_MDRD_) and CKD-EPI (eGFR_CKD-EPI_) formulas.

**Results:**

Forty-five patients continued CSA and 96 patients were converted to TAC with a median follow up of 24 months. Baseline demographics (except for recipient age) including native kidney disease, transplant characteristics, kidney graft function, medication use and comorbid conditions did not differ between groups. In respect to long-term renal graft function, linear mixed models showed significantly improved eGFR trajectories (eGFR_MDRD_: p<0.001, eGFR_CKD-EPI_: p<0.001) in the TAC versus CSA group over 24 months of follow up. Estimated eGFR_CKD-EPI_ group differences between TAC and CSA were −3.49 (p = 0.019) at 3 months, −5.50 (p<0.001) at 12 months, and −4.48 ml/min/1.73m^2^ (p = 0.003) at 24 months of follow up. Baseline eGFR was a significant predictor of eGFR trajectories (eGFR_MDRD_: p<0.001, eGFR_CKD-EPI_: p<0.001). Significant effects for randomization group were evident despite short-term trough levels in the supratherapeutic range (27% (n = 26) of TAC patients at week one). Median TAC trough levels were within target range at week 4 after conversion.

**Conclusion:**

Conversion of CSA treated kidney transplant recipients with stable graft function to TAC (target 5–8 ng/ml) showed significantly improved long-term eGFR trajectories when compared to CSA maintenance (target 70–150 ng/ml).

**Trial Registration:**

ClinicalTrials.gov NCT00182559 EudraCT identifier: 2004-004209-98

## Introduction

Tacrolimus (TAC) versus ciclosporin A (CSA) as initial immunosuppression in kidney transplant recipients has shown improved graft function, reduced acute rejection episodes and reduced kidney graft loss [1,2]. Promising results of de-novo studies have led to conversion studies describing effects on renal function in stable grafts [3,4] and grafts with chronic allograft lesions [5–7]. Reported effects on renal function in stable grafts were mixed with one group reporting a decline in measured creatinine clearance for patients maintained on CSA [3] versus another group reporting a more favourable median change from baseline in estimated creatinine clearance for patients converted to reduced TAC [4]. Both studies were randomized and controlled but differed in respect to targeted CNI trough levels (TAC target: 5–8 ng/ml versus 3.5–5.9 ng/ml in reduced TAC versus 6.0–8.9 ng/ml in standard TAC arm; CSA target: 50–200 ng/ml versus 50–250 ng/ml), statistical approach and power. In respect to assessing renal function, it has been suggested that estimations of GFR via MDRD (eGFR_MDRD_) and CKD-EPI (eGFR_CKD-EPI_) perform with less bias and greater accuracy than equations based on measured or estimated creatinine clearance [8,9].

CNI conversion in stable kidney transplant recipients remains of interest as calcineurin inhibitor nephrotoxicity has been associated with changes in renal function [10], implicated in the unchanged problem of long term graft attrition [11], and sufficiently powered randomized controlled conversion trials remain sparse.

This is a secondary analysis of a previously published randomized controlled trial focusing on endothelial progenitor cells in long-term kidney transplant recipients with stable graft function converted from CSA to TAC [12]. We briefly reported on renal function (secondary endpoint) at 24 months of follow-up in the published study. However, statistical power was not high enough to show between group differences below 10 ml/min/1.73m^2^. As clinically relevant differences between groups might be much lower in long-term kidney transplant recipients, we aimed at studying renal function trajectory after randomization (multiple renal function measurements per patient over time) using linear mixed models, which provide effect estimates on both slope and baseline level of GFR [13], and offer increased statistical power for eGFR comparison.

## Subjects and Methods

### Study Design

Study design including participants, interventions, outcome measures, sample size calculations and randomization have been described in detail earlier [12]. Briefly, the Vienna PEP study was a 2:1 randomized, parallel group, open-label, prospective trial comparing two different immunosuppressive regimens in kidney transplant recipients. The TAC group was converted from ciclosporin to tacrolimus at a target trough level of 5–8 ng/ml in combination with or without mycophenolate/mycophenolic acid (MMF/EC-MPA) and with or without steroids. The CSA group was maintained on ciclosporin in combination with or without MMF/EC-MPA and with or without steroids and without a change in target trough level of 70–150 ng/ml. Patients were recruited between April 2005 and May 2007 and followed up for 24 months after conversion, with study specific visits at baseline, at 3, at 12, and at 24 months. Patients converted to tacrolimus had additional visits at week 1, 2, 4 and 8 for dose adjustments. Evaluations at these time-points included routine clinical assessments, dosing and through levels of CSA and TAC, graft function, cardiovascular disease risk factors, and safety parameters.

### Patient Cohort

Long-term kidney transplant recipients (n = 141) with stable graft function were recruited between April 2005 and May 2007 at Medical University Vienna (Division of Nephrology and Dialysis, Department of Medicine III, Medical University of Vienna) and followed up for 24 months. For the last patient recruited, follow-up ended in May 2009. We recruited recipients of deceased- or living-donor kidney transplants, at least 6 months after transplantation, with stable graft function (eGFR_MDRD4_ ≥ 30ml/min/1.73m^2^ within four weeks before study entry and no biopsy-proven acute rejection within 3 months before study entry), and without a history of cardiovascular events within 3 months before study entry. Patients with recurrence of primary kidney disease or known malignancy were not included. The full list of in- and exclusion criteria was shown in tabular form earlier [12].

### Study Medication

After randomization TAC was administered orally with 0.1 mg/kg bodyweight, divided into two doses, as suggested in the package insert. The dose was adjusted to attain whole blood trough target concentrations of 5 to 8 ng/mL. Supratherapeutic TAC through levels were defined as above 20 ng/ml. In patients converted to TAC, the MMF/EC-MPA dose was reduced to account for increased enterohepatic recirculation of glucuronidated mycophenolic acid [14]. Pre-conversion MMF (EC-MPA) doses of 2g/day (1440mg/day) and 1.5g/day (1080mg/day) were reduced to 1.5g/day (1080mg/day) and 1g/day (720mg/day), respectively. In patients maintained on CSA, the MMF/EC-MPA dose remained unaltered. Corticosteroids remained unchanged in both groups.

### Laboratory Procedures

Parameters were measured using routine procedures at the Clinical Institute for Laboratory Medicine at the Medical University of Vienna. Serum creatinine was measured using a kinetic alkaline picrate reaction (Jaffe reaction) and was not standardized to isotope dilution mass spectrometry. Estimated GFR (eGFR) was calculated with the published equations for four-variable MDRD [15] and CKD-EPI [9]. As eGFR_CKD-EPI_ is expressed for standardized creatinine, values were reduced by 5% for this equation, the calibration of the Modification of Diet in Renal Disease study laboratory [16], mirroring studies in chronic kidney disease [17,18].

### Statistics

In descriptive analyses, continuous variables were expressed as mean±standard deviation (SD) or median (Q1-Q3). Categorical variables were expressed as percentages and the differences between proportions were compared using chi-square tests. T-tests or Mann-Whitney rank-sum tests were used to test for differences between groups. Differences between renal function at baseline, week 1, 2, 4 and 8 within the TAC group were compared using the Friedman test for repeated measures. Linear mixed models with a spatial Gaussian time-series-type covariance structure were calculated [13] for eGFR_MDRD_ and eGFR_CKD-EPI_: Explanatory variables were eGFR_MDRD_ or eGFR_CKD-EPI_ at randomisation (i.e. before conversion), randomization group (CSA versus TAC), categorical time (3, 12 and 24 months) and the pair-wise interaction of randomization group and time; dependent variables were either eGFR_MDRD_ or eGFR_CKD-EPI_ (measurements at 3, 12 and 24 months). An appropriate covariance structure between subjects as well as within subjects was selected based on the AIC. The model fit was evaluated by visualizing studentized residuals, the linearity assumption of eGFR_MDRD_ or eGFR_CKD-EPI_ at randomisation was checked with models using B-splines for the eGFR measurements at randomisation, and influence of individual subjects on parameter estimates was evaluated. A post-hoc power analysis yielded a power of 81% (two-group t-test, degrees of freedom are the effective degrees of freedom from the repeated measures ANOVA) to detect a mean difference in eGFR of 4.5 ml/min/1.73m^2^ with a standard deviation of 20 ml/min/1.73m^2^ and a significance level of 0.05. Calculations were done using R 3.0.2 [19] and SAS 9.3 [20].

### Ethics

The protocol was reviewed and approved by the institutional review board on human research at the Medical University Vienna on December 22, 2004. The study was conducted according to the Declaration of Helsinki. The clinical and research activities reported are consistent with the principles outlined in the ‘Declaration of Istanbul on Organ Trafficking and Transplant Tourism’. Written informed consent was obtained according to International Conference on Harmonization-Good Clinical Practice. The trial was registered at EudraCT (2004-004209-98; https://www.clinicaltrialsregister.eu/ctr-search/trial/2004-004209-98/AT) on March 11, 2005 (before starting patient recruitment). For completeness, this registration was followed by a registration at clinicaltrials.gov (NCT00182559; https://clinicaltrials.gov/ct2/show/NCT00182559) on September 10th 2005 (after starting patient recruitment). The authors confirm that all ongoing and related trials for this drug/intervention are registered.

## Results

### Patient Demographics

**Fig 1:**
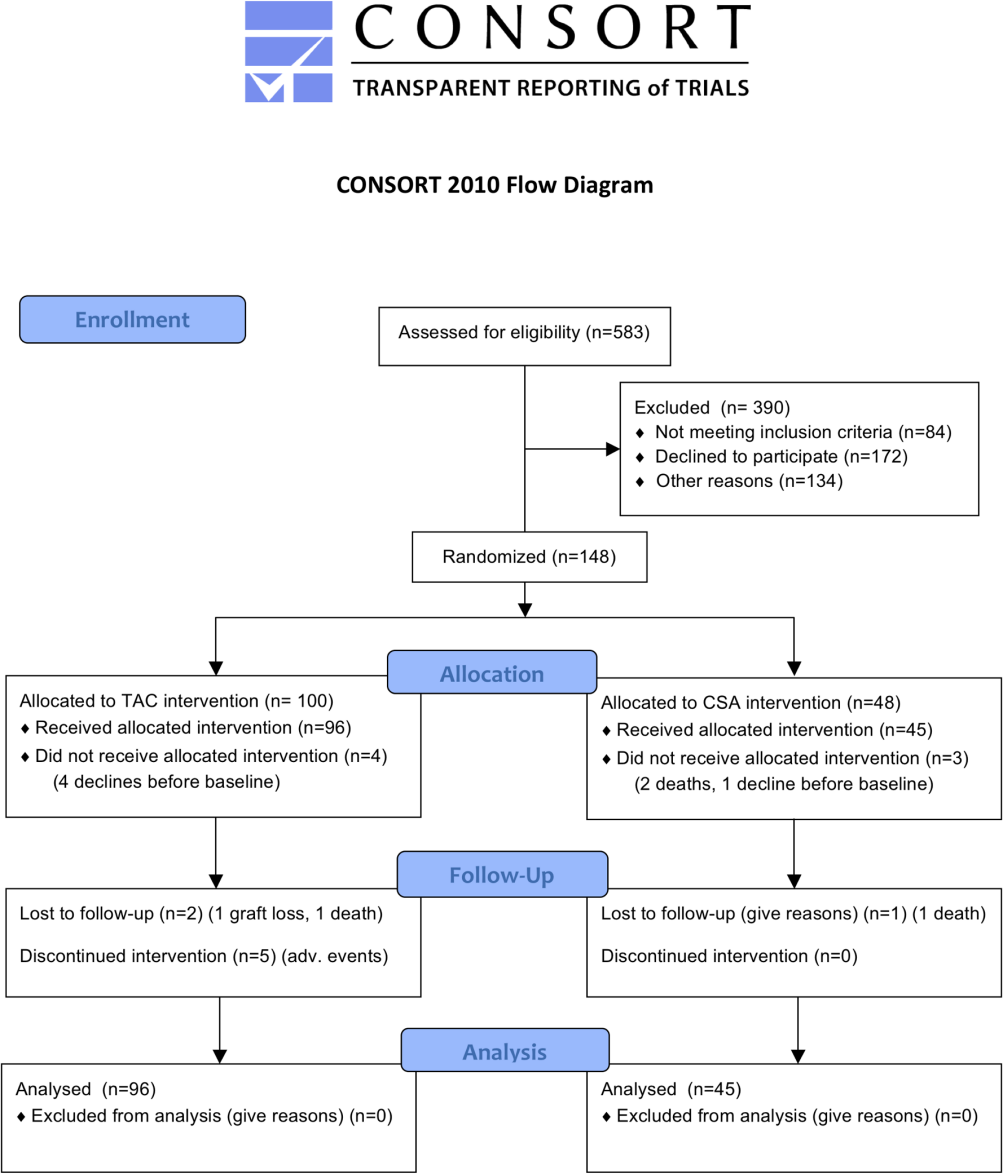
Consort flow diagram.

One hundred forty-eight individuals were randomized and 141 were included in the intention-to- treat (ITT) analysis (Fig 1). Baseline demographics relevant to kidney function and allograft survival for CSA and TAC groups are shown in Table 1. The majority of patients were of European descent and male. Median age was higher in the CSA group (62 versus 53 years; p = 0.008). Native kidney disease, medication use and comorbid conditions such as smoking status were similar in both treatment arms. Transplant related variables such as baseline eGFR, HLA mismatch, donor age, proportion of deceased versus living and living-related grafts and proportion of patients receiving each concomitant immunosuppressive regimen were also comparable between treatment arms.

**Table 1:** Baseline Demographics for Patients Randomized to CSA and TAC.

	CSA (n = 45)	TAC (n = 96)	
Age (years), median (Q1-Q3)	61.77 (50.81–67.20)	53.22 (44.92–62.96)	**p = 0.008**
Male Sex, n (%)	33 (73)	62 (65)	p = 0.40
**Race/Ethnicity**			
European, n (%)	43 (96)	93 (97)	p = 0.27
African, n (%)	2 (4)	1 (1)	
Asian, n (%)	0 (0)	2 (2)	
Height (cm), mean±SD	173.2±7.7	170.4±8.8	p = 0.06
Weight (kg), median (Q1-Q3)	78.0 (70.0–85.0)	75.9 (65.8–83.5)	p = 0.22
Serum Creatinine (mg/dl), mean±SD	1.45 (1.26–1.78)	1.56 (1.27–1.88)	p = 0.58
eGFR_MDRD_ (ml/min/1.73m2), mean±SD	49.79 ± 15.60	47.37 ± 13.78	p = 0.38
eGFR_CKD-EPI_ (ml/min/1.73m2), mean±SD	52.10 ± 16.73	50.72 ± 15.61	p = 0.64
**Native kidney disease**			
Polycystic kidney disease, n (%)	9 (20)	9 (9)	p = 0.44
Diabetic nephropathy, n (%)	3 (7)	8 (8)	
Glomerular disease, n (%)	14 (31)	28 (29)	
Tubulointerstitial disease, n (%)	5 (11)	13 (14)	
Vascular, n (%)	5 (11)	11 (12)	
Other, n (%)	0 (0)	6 (6)	
Unknown, n (%)	9 (20)	21 (22)	
**Donor:**			
Donor Age (years), median (Q1-Q3)	46.0 (31.5–56.0)	45.0 (30.0–54.0)	p = 0.73
Donor Male Sex, n (%)	28 (62)	54 (56)	p = 0.40
**Graft, n (%)**			
Living unrelated	4 (9)	4 (4)	p = 0.24
Living related	2 (4)	11 (12)	
Deceased	39 (87)	81 (84)	
HLA MM (A+B+DR)	2.6±1.5	2.6±1.5	p = 0.97
PRA > 5%, n (%)	5 (11)	22 (23)	
Graft vintage (years), median (Q1-Q3)	6.8 (4.7–12.8)	6.1 (2.9–11.3)	p = 0.25
Urine Albumin-Creatinin-Ratio (mg/g), median (Q1-Q3)	7 (19–85)	26 (8–116)	p = 0.52
Smoker, n (%)	4 (9)	15 (16)	p = 0.41
Ex-Smoker, n (%)	16 (36)	35 (36)	p = 1
DM			
Type I	2 (4)	3 (3)	p = 0.26
Type II	4 (9)	19 (20)	
Antihypertensive medication number, median (Q1-Q3)	3 (2–4)	2 (2–3)	p = 0.11
Specific medications			
ACEI, n (%)	19 (42)	41 (43)	p = 1
ARB, n (%)	16 (36)	33 (34)	p = 1
Statin, n (%)	22 (49)	51 (53)	p = 0.77
Fibrates, n (%)	0 (0)	4 (4)	p = 0.40
ESA, n (%)	9 (20)	20 (21)	p = 1
**Immunosuppression at study entry**			
CSA, n (%)	3 (7)	5 (5)	p = 0.54
CSA+Steroids, n (%)	9 (20)	12 (13)	
CSA+MMF/EC-MPA, n (%)	13 (29)	25 (26)	
CSA+MMF/EC-MPA+Steroids, n (%)	20 (44)	54 (56)	
CSA daily dose (mg), median (Q1-Q3)	150 (125–200)	150 (125–175)	p = 0.95
CSA Trough Level (ng/ml), median (Q1-Q3)	78 (63–95)	72 (63–95)	p = 0.83
MMF/EC-MPA daily dose (mg), median (Q1-Q3)	1500 (1000–2000)	1500 (1000–2000)	p = 0.49
Steroids daily dose (mg), median (Q1-Q3)	2.5 (1.25–5)	2.5 (1.25–5)	p = 0.76

### Follow-Up

Graft loss was experienced by one patient in the TAC group shortly before the patient finished the main study (22 months of follow-up, no biopsy available; clinical course suggests chronic failure). One patient died in each group during the study period (cause: CSA: hepatocellular carcinoma, TAC: unknown). During follow-up, four patients in the TAC group were reconverted to CSA due to pruritus (n = 2) and alopecia (n = 2) and one patient in the TAC group was converted to sirolimus due to anogenital papillomatosis.

### Immunosuppressant Exposure

**Fig 2:**
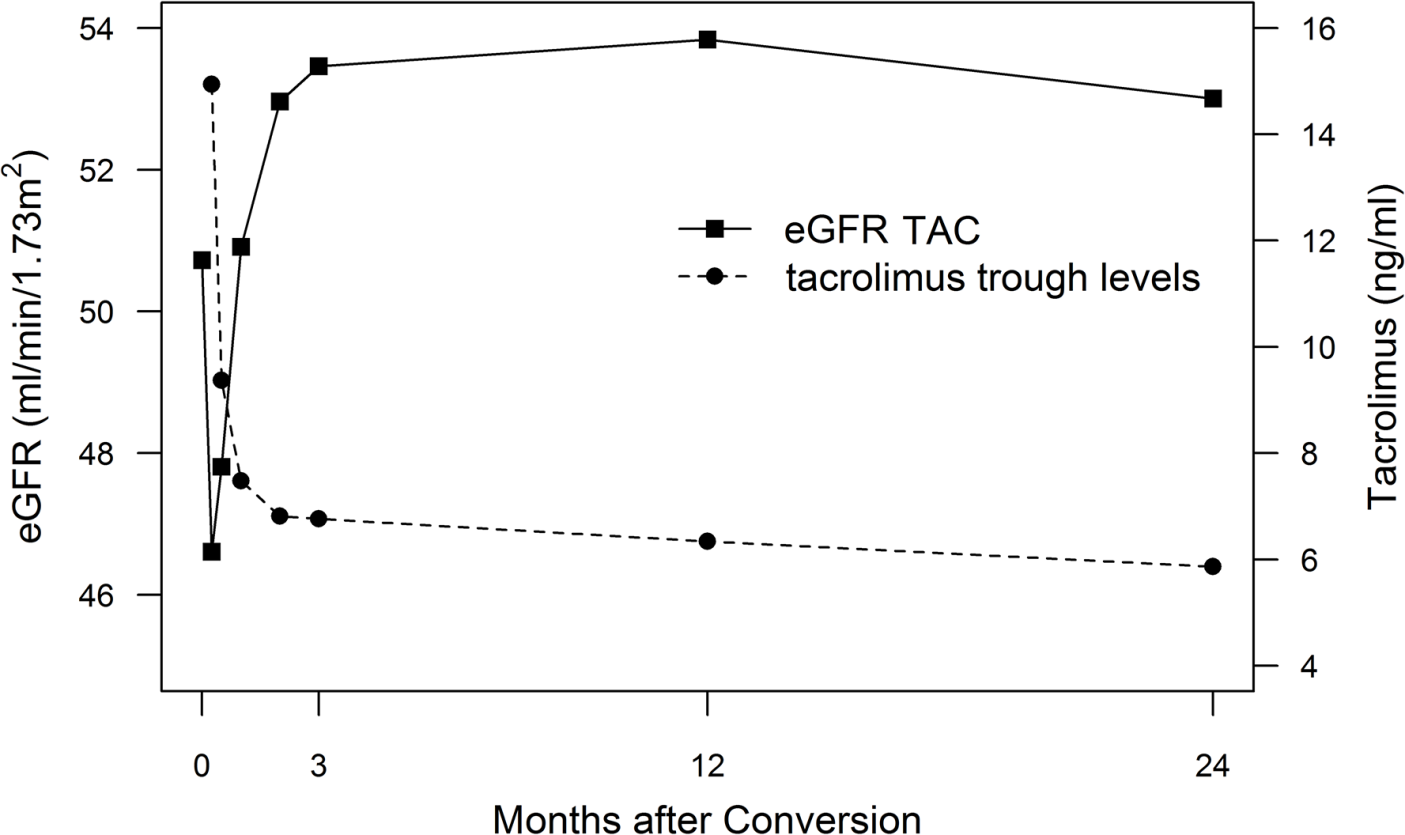
TAC group eGFR and trough level fluctuations. Mean eGFR_CKD-EPI_ (solid line) and trough levels (dashed line) for the TAC study group at baseline, weeks 1, 2, 4, 8, and 3, 12 and 24 months after conversion.

**Fig 3:**
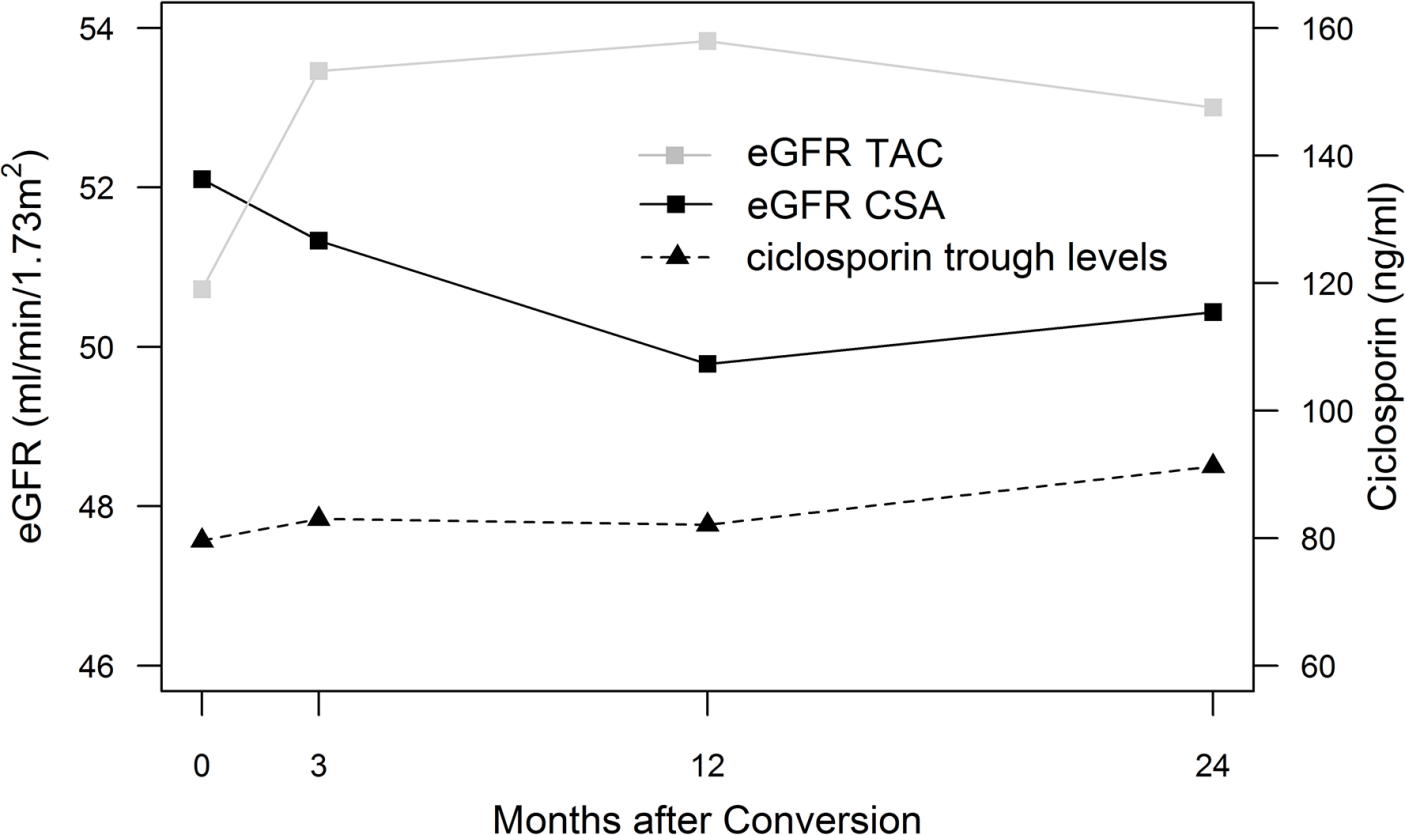
CSA and TAC group eGFR courses compared. Mean eGFR_CKD-EPI_ for the CSA (solid black line) and TAC study group (solid grey line) at baseline, 3, 12 and 24 months after conversion. Ciclosporin trough levels (dashed line) at the same timepoints.

**Fig 4:**
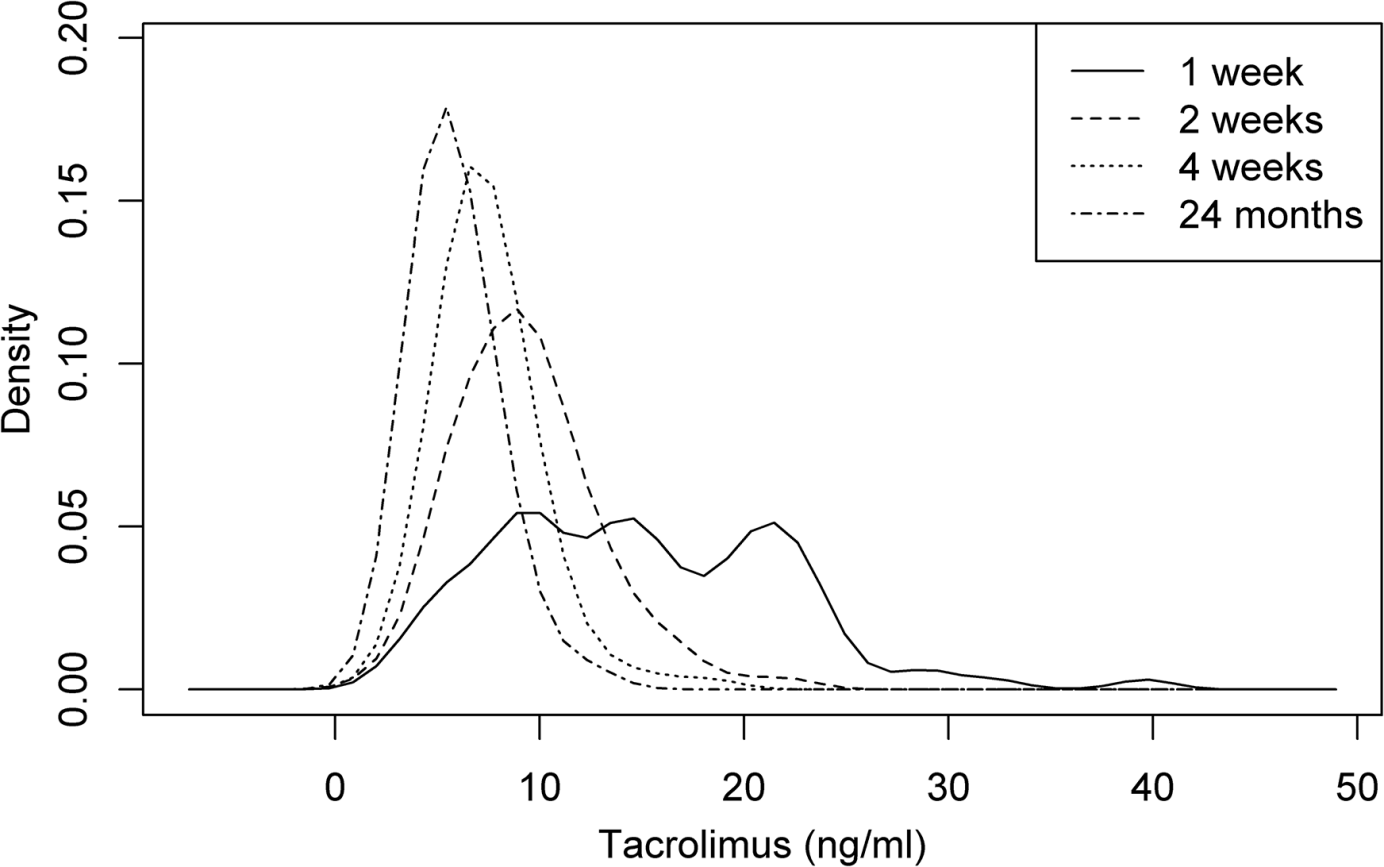
TAC trough level distributions at different follow-up timepoints. Tacrolimus trough level distributions at 1, 2 and 4 weeks, as well as, 24 months after conversion shown as density plots.

Figs 2 and 3 show median trough level courses for TAC and CSA study groups. For the TAC group additional measurements are shown in Fig 2 for week 1, 2, 4 and 8 after conversion. Using a conversion dose of 0.1 mg/kg TAC, trough levels were highest (14.2 (9.4–20.5) ng/ml) one week after conversion, with 27% (n = 26) of TAC patients being in the supratherapeutic range (>20 ng/ml). Median trough levels were titrated to target range until week 4 (7.2 (6.0–8.6) ng/ml). At 24 months TAC trough levels were at a median of 5.5 (4.5–7.0) ng/ml with a median dose of 0.04 (0.03–0.06) mg/kg/day. TAC trough level distributions at 1, 2 and 4 weeks as well as 24 months after conversion are shown as density plots in Fig 4. CSA trough levels were not measured at week 1, 2, 4 and 8 for patients maintained on CSA. CSA trough levels were at a median of 89.0 (68.3–108.2) ng/ml with a median dose of 1.68 (1.45–2.52) mg/kg/day at 24 months of follow-up.

### Short-Term Renal Graft Function

Fig 2 shows the short- to long-term (mean) eGFR_CKD-EPI_ course for the TAC study group, including measurements for week 1, 2, 4 and 8 after conversion. In the TAC group eGFR was lowest at week 1 (eGFR_MDRD_: 43.62 ± 12.93 ml/min/1.73m^2^; eGFR_CKD-EPI_: 46.61 ± 14.78 ml/min/1.73m^2^) and changed significantly between baseline, week 1, 2, 4 and 8 (Friedman: p<0.001). eGFR_CKD-EPI_ was not measured at week 1, 2, 4 and 8 for patients maintained on CSA.

### Long Term Renal Graft Function

Fig 3 shows mean eGFR_CKD-EPI_ (trajectories) for both study groups at baseline, 3, 12 and 24 months. Over 24 months of follow-up linear mixed models for eGFR_MDRD_ and eGFR_CKD-EPI_ showed significant effects for randomization group (eGFR_MDRD_: p<0.001, eGFR_CKD-EPI_: p<0.001) and baseline eGFR (eGFR_MDRD_: p<0.001, eGFR_CKD-EPI_: p<0.001). Effect comparison between CSA and TAC showed significantly higher eGFR estimates for the TAC group at all timepoints (3, 12, and 24 months). eGFR estimates as well as estimated group differences between CSA and TAC (for both eGFR_MDRD_ and eGFR_CKD-EPI_) are shown in Table 2. Neither time (follow-up up to 24 months) (eGFR_MDRD_: p = 0.618, eGFR_CKD-EPI_: p = 0.582) nor the pair-wise interaction between randomization group and time (eGFR_MDRD_: p = 0.461, eGFR_CKD-EPI_: p = 0.490) showed statistically significant effects.

**Table 2:** Effect Comparison in linear mixed models for eGFR_MDRD_ and eGFR_CKD-EPI_.

eGFR_MDRD_	**Estimate CSA**	**Estimate TAC**	**Difference**
**3 months**	47.59 (SE 1.12)	50.62 (SE 0.77)	−3.04 (SE 1.36, p = 0.026)
**12 months**	46.08 (SE 1.12)	51.05 (SE 0.77)	−4.98 (SE 1.36, p<0.001)
**24 months**	46.41 (SE 1.13)	50.35 (SE 0.77)	−3.94 (SE 1.37, p = 0.004)
eGFR_CKD-EPI_	**Estimate CSA**	**Estimate TAC**	**Difference**
**3 months**	50,43 (SE 1.22)	53.91 (SE 0.84)	−3.49 (SE 1.48, p = 0.019)
**12 months**	48.88 (SE 1.22)	54.38 (SE 0.84)	−5.50 (SE 1.48, p<0.001)
**24 months**	49.08 (SE 1.23)	53.55 (SE 0.84)	−4.48 (SE 1.49, p = 0.003)

## Discussion

In this secondary analysis of a large RCT, conversion of CSA treated kidney transplant recipients with stable graft function to TAC showed significantly improved eGFR_MDRD_ and eGFR_CKD-EPI_ trajectories when compared to CSA maintenance over 24 months of follow up. Improved trajectories were seen despite short term TAC trough levels in the supratherapeutic range.

As creatinine clearance (estimated by Cockcroft-Gault) is no longer recommended in current guidelines (KDIGO Evaluation and Management of CKD) for its lack of traceability to standardized creatinine and its overestimation of the true level of GFR with progressing renal disease, we reported both eGFR_MDRD_ and eGFR_CKD-EPI_. eGFR_MDRD_ has been shown to predict GFR more accurately than equations based on measured or estimated creatinine clearance [8]. eGFR_CKD-EPI_ was shown to perform even better than eGFR_MDRD_, especially at higher GFR, with less bias and greater accuracy [9] as eGFR_MDRD_ was developed using a study cohort limited to individuals with chronic kidney disease [21]. With mean baseline eGFR_MDRD_ values of 49.8 in the CSA versus 47.4 ml/min/1.73m2 in the TAC group, eGFR_CKD-EPI_ thus promised to offer the best eGFR estimates in our cohort.

From a statistical standpoint we chose to use linear mixed models, as they are a preferred method to investigate risk factors associated with renal function trajectories. Mixed models of eGFR trajectory offer a vast improvement over classical between group comparison of eGFR, as they provide effect estimates on both slope and baseline level of GFR unaffected by patient dropout. [13] Using linear mixed models versus seperate direct group comparisons at specific timepoints using t-tests we were also able to significantly increase our studies statistical power to detect a mean difference in eGFR between groups (4.5 versus 10 ml/min/1.73m^2^).

Two comparable studies have been performed in stable kidney transplant recipients. The Optima Trial randomly assigned 328 stable transplant recipients to 1) continue on CSA with a target trough level of 50–250 ng/ml, 2) convert to reduced dose TAC with a target trough level of 3–5.9 ng/ml, or 3) convert to standard dose TAC with a target trough level of 6–8.9 ng/ml. The authors reported a more favorable median change of estimated creatinine clearance and serum creatinine from baseline to 12 months of follow-up for the reduced TAC group compared with the CSA group. No statistically significant difference in median change from baseline was observed for standard dose TAC versus CSA. Trough levels showed a large overlap between reduced and standard dose TAC arms. TAC trough levels in our study were closer to the standard dose arm of this trial. Median CSA trough levels at 12 months were about 30% higher in this trial than in our trial. [4]

A second trial in stable kidney transplant recipients by Artz et al. was similar to our trial in terms of baseline renal function and graft vintage and also featured a follow up of 2 years. At 24 months creatinine clearance was significantly lower in the CSA group (target trough level: 50–200 ng/ml) using ANCOVA with randomization group and baseline value as independent factors. Additionally, in contrast to the TAC group (target trough level: 5–8 ng/ml), a significant change of creatinine clearance was seen within the CSA group while the TAC group remained stable over the time of follow-up. Interestingly, study medication was withdrawn in 25% of each arm, mostly because of side effects. In our study 5 out of 96 patients (5%) in the TAC group were (re-)converted to CSA or sirolimus during follow-up due to side effects. CSA levels were about 30% higher than in our study. Mean tacrolimus trough levels were 6.6 ± 1.8 ng/mL at 2 years after randomization and thus somewhat higher than in our cohort. [3]

Considering the nephrotoxic nature of both CSA and TAC [22] the varying study results seen in terms of change in renal function after randomization might be affected by achieved CSA and TAC trough levels. However, as CSA levels were about 30% higher in the published cohorts and low dose CSA has been associated with improved kidney graft function [23], it seems unlikely that estimated eGFR group differences between CSA and TAC in our cohort would have been lower with higher CSA doses. Furthermore, our cohorts trough targets are in line with current de-novo trough targets. In de-novo patients low dose tacrolimus (target: 3–7 ng/ml) has been associated with higher GFR, lower biopsy-proven acute rejection episodes and higher allograft survival when compared to standard (100–200 ng/ml) and low-dose CSA (50–100 ng/ml) [1].

Acute CNI neprotoxicity has been described as acute hemodynamically mediated renal dysfunction and regarded as a reversible phenomenon [22]. Although even short term side effects of CNI overdosing seem dispensable (27% of our studies TAC patients were in the supratherapeutic trough level range at week 1), our data adds to the published literature by suggesting that long term kidney transplant recipients might non the less benefit from conversion to TAC in terms of eGFR trajectory.

Our studies limitations include not beeing powered for harder endpoints such as graft loss, as well as being performed prior to the introduction of isotope dilution mass spectrometry. Nevertheless, we provide strong evidence for a significant improvement in renal function trajectory using a large randomized controlled trial, more accurate methods for estimation of renal function and a state of the art statistical analysis.

In conclusion, conversion of CSA treated kidney transplant recipients with stable graft function to TAC (target 5–8 ng/ml) showed significantly improved long-term eGFR trajectories when compared to CSA maintenance (target 70–150 ng/ml).

## Supporting Information

S1 CONSORT Checklist This is the CONSORT Checklist for our study. (DOC)

S1 Dataset Minimal Data Set. This is the minimal data set underlying the findings in our study manuscript.
(XLSX)

S1 Protocol Study Protocol. This is the protocol for our study. (DOC)

S1 Table Multivariable Data Analysis Results. Additional information about the two models from Table 2.
(DOCX)
