# Supplementary material for: Late Conversion of Kidney Transplant Recipients from Ciclosporin to Tacrolimus Improves Graft Function: Results from a Randomized Controlled Trial
Source: PLoS One. 2015 Aug 13;10(8):e0135674. doi: 10.1371/journal.pone.0135674 (PMC4535983; doi:10.1371/journal.pone.0135674)
Supplement: S1 Protocol — (DOC) [file pone.0135674.s003.doc]

**The Vienna Prograf and Endothelial Progenitor Cell**

**(Vienna PEP) Study**

Division of Nephrology and Dialysis, Department of Medicine III

Medical University Vienna, Austria

Short title: The Vienna PEP study

Correspondence:

Gere Sunder-Plassmann, M.D.

Division of Nephrology and Dialysis

Department of Medicine III, Medical University Vienna

Währinger Gürtel 18-20, A-1090 Vienna, Austria

phone: +43-1-40400-4391

fax: +43-1-40400-4392

mail: gere.sunder-plassmann@meduniwien.ac.at

**Hypothesis**

Endothelial progenitor cells are bone marrow-derived cells that are capable of maturating into the specific cell type that constitutes the lining for blood vessels in the body . Initially, it was shown that mononuclear blood cells from healthy individuals had the potential to develop an endothelial cell phenotype in vitro and to incorporate these cells into blood vessels . These cells expressed CD34, and vascular endothelial growth factor receptor-2 (VEGFR-2, also referred to as KDR), two surface antigens that are present on hematopoietic stem cells. Peichev et al. later identified a population of CD133+ (also referred to as AC 133) cells among CD34+/VEGFR-2+ cells in the peripheral blood, which also acquire an endothelial phenotype, but lose CD133 after differentiation . There is some controversy in the literature concerning the exact phenotype of EPCs . For instance, CD34- cells from the monocyte/macrophage lineage were also shown to be able to differentiate into functional endothelial cells . It is the current understanding that EPCs play a crucial role in rapid re-endothelialization after injury and in neovascularization . Indeed, therapeutic angiogenesis and vasculogenesis using autologous transplantation of mononuclear bone marrow cells or ex vivo expanded EPCs improved tissue injury and organ function following ischemia of a limb or of the heart in humans.

Stimulatory or inhibitory factors modulate EPC levels, and may thus affect the vascular repair capacity . There is indication that the number and functional activity of EPCs may be associated with presence or absence of several cardiovascular disease risk factors, including smoking, hypertension, diabetes, or hyperlipidemia in patients with or without coronary artery disease . Hill et al have postulated the number of EPCs to be a surrogate biologic marker for vascular function and cumulative cardiovascular risk . EPCs have also been shown to be decreased in patients with end-stage renal disease . Furthermore, statin use or erythropoietic therapy appear to mobilize EPCs from the bone marrow and enhance the functional capabilities of these cells.

In addition to hypertension, diabetes, hyperlipidemia, and smoking , as well as other non-traditional risk factors such as elevated C-reactive protein, homocysteine, or Lp(a), or reduced renal function , depletion of EPCs in the peripheral circulation may represent another important explanation for the excess cardiovascular morbidity and mortality of kidney transplant recipients .

In this context, the potential association of immunosuppressive therapy with EPCs in kidney transplant recipients deserves special consideration. The use of tacrolimus was associated with a more favorable cardiovascular risk factor profile in terms of improved blood pressure and lipid levels in kidney transplant recipients compared to cyclosporine users . Therefore, one can speculate whether tacrolimus users might have greater EPC counts compared to patients treated with cyclosporine. However, studies of EPCs in kidney transplant recipients are scarce.

In a pilot study we cross-sectionally studied EPC counts in 90 stable, middle-aged kidney transplant recipients . Using univariate and multivariate linear regression, we examined the associations between EPC counts and traditional cardiovascular disease risk factors (hypertension, diabetes, hyperlipidemia, smoking), kidney function, and different immunosuppressive agents, amongst others. From multivariate analyses, we found independent inverse associations between EPC counts and body mass index and systolic blood pressure. Statin use was associated with greater EPC counts, while patients receiving azathioprine had lower EPC counts. These findings raised the hypothesis whether EPCs are responsible, at least in part, for the well-established associations between these factors and cardiovascular outcomes. In this study, neither tacrolimus versus cyclosporine use, nor the trough levels of either drug were associated with EPCs. This lack of association may be related to the cross-sectional design of this study, where most of the tacrolimus users were previously converted from cyclosporine because of side effects, or acute rejection episodes. Therefore, randomized trials are necessary to clarify this important issue.

**METHODOLOGY**

**STUDY OBJECTIVES**

The study objectives are (1) to test the hypothesis that conversion from cyclosporin A to tacrolimus based immunosuppressive therapy increases EPC counts in stable long-term kidney transplant recipients, (2) to examine graft function, (3) to determine humoral alloreactivity, (4) to study pharmocokinetic and pharmacogenomic aspects of tacrolimus based therapy, and (5) to document changes in the cardiovascular disease risk profile and the safety after conversion from cyclosporin A to tacrolimus.

**STUDY DESIGN**

The Vienna PEP study is a 2:1 randomized, parallel group, open-label, prospective trial comparing two different immunosuppressive regimens in kidney transplant recipients (Group A: Conversion from cyclosporin A to tacrolimus at a target trough level of 5-8 ng/ml in combination with/without MMF and w/wo steroids. Group B: Maintain cyclosporine A in combination with/without MMF and w/wo steroids without change in target trough level (usually 70 - 150 ng/ml), **Figure 1**).

The protocol was reviewed and approved by the institutional review board on human research at the Medical University Vienna.

Patients will be followed up for 24 months after conversion, with study specific visits at baseline, at 3, at 12, and at 24 months (patients converted to tacrolimus had additional visits at week 1 and 2 for dose adjustments). Evaluations at these time-points include standard clinical assessments as well as analyses of EPCs, dosing and through levels of CSA and TAC, graft function, PRAs, CVD risk factors, and safety.

**PATIENT POPULATION**

Long-term kidney transplant recipients with stable graft function and no immediate history of cardiovascular disease events from the transplant clinic of the Division of Nephrology and Dialysis, Department of Medicine III, Medical University Vienna, were included in this study. The inclusion and exclusion criteria are indicated in **Table 1**.

**ENROLLMENT AND RANDOMIZATION**

Patients who were deemed eligible for study inclusion, who did not exhibit any exclusion criteria, and who have provided written informed consent were randomly allocated to one of the two treatment arms (stratification by statin use and/or erythropoietin use). Logistically, the attending physician (study ccordinator) contacted the randomization center at the Division of Pharmacoepidemiology and Pharmacoeconomics, Brigham and Women’s Hospital, Harvard Medical School (Boston,MA, U.S.A.) via email, where a random number generator will allocate patients to continuing treatment at probability P=0.333 vs. switching to tacrolimus at probability P=0.667. The result of the random process will be communicated back to the attending physician via email, and documented both in the medical record as well as at the randomization center.

**STUDY MEDICATION**

**Immunosuppressive therapy**

At the time of study entry (baseline visit), all patients were receiving cyclosporine A-based immunosuppression in combination with/without MMF and w/wo steroids. Patients randomized to tacrolimus will discontinue cyclosporine and initiate tacrolimus therapy within 12 hours of the last dose of cyclosporine.

Tacrolimus was given orally with 0.1 mg/kg BW in two divided doses. The dose was adjusted to attain target whole blood trough concentrations of 5-8 ng/ml.

In patiente converted to tacrolimus, MMF was reduced by 25-50% because Cyclosporine users need higher MMF doses. In patients maintained on a cyclosporine based immunosuppression, the MMF dose was not changed. The dose of corticosteroids was not changed in either group.

**Diagnosis and treatment of rejection episodes**

Episodes of rejection must be biopsy-confirmed within 24 hours of initiating treatment. All biopsy specimens will be graded using the Banff criteria. Allograft rejection may be treated with an increase in dose and trough level of tacrolimus, corticosteroids, or antilymphocyte therapy. Patients treated with anti-lymphocyte therapy may use Pneumocystis carinii prophylaxis and CMV prophylaxis. The need to change immunosuppressive therapy as treatment for rejection will be considered an outcome of the study. However, these patients will continue to be followed for the remainder of the study.

**ENDPOINTS**

**Primary endpoint**

The primary end-point is to determine the effect of conversion from cyclosporine to tacrolimus-based therapy on EPC count at 24 months.

**Secondary endpoints**

Secondary endpoints to be assessed in this study include the following:

(1) Renal function as assessed by cystatin C measurement, serum creatinine, and serum creatinine based equations to estimate creatinine clearance or GFR. The incidence and severity of biopsy-confirmed acute rejection will be assessed at months 12 and 24.

(2) Alloreactivity against a panel of HLA antigens (panel reactive antibodies, PRA) as a surrogate marker of humoral alloreactivity against the transplanted organ. Presence of PRA will be evaluated using flow cytometry analysis.

(3) Pharmcokinetic and pharmacogenetic evaluations including the association of SNPs in MDR1 and CYP450 with concentration to dose ratios of immunosuppressants at the specific study visits.

(4) Changes in risk factors for cardiovascular disease outcomes (serum lipids, blood pressure, diabetes mellitus, serum C-reactive protein, body mass index, Framingham risk score).

(5) Safety will be adressed according to indicators given in **Table 2**.

**STATISTICAL ANALYSES**

**Sample size**

The sample size calculation is based on the results of a pilot study of 90 stable kidney graft recipients, where the distribution of EPC counts was found to be strongly skewed to the right. Therefore, we used logarithmical transformation to normalize the distribution of the data. The mean lnEPC count was 3.6 ± 1.0 per high power field. Assuming an increase of 15% in lnEPC count after conversion from cyclosporine to tacrolimus (asimilar effect was observed for statin users versus non-users in our pilot study) that persists for at least 2 years, and using a 2:1 randomization, 41 patients are needed in the cyclosporine group, and 82 in the tacrolimus group (type I error 0.05, power 80%). Accounting for an annual rate of graft loss and death of 5%, and an annual rate of drop out related to further changes in the immunosuppressive protocol of 5%, 49 patients are needed for the cyclosporine group, and 99 patients are needed for the tacrolimus group. The paired t-test will be used to compare lnEPC between the treatment groups at 3, 12 and 24 months post-randomization.

**LABORATORY ANALYSES**

Blood and urine chemistry, full blood counts, and measurement of through levels of cyclosporin A and tacrolimus were performed in the Clinical Laboratory at the Medical University of Vienna

**Endothelial progenitor cells**

This study of EPCs will include (1) an analysis of EPCs grown from peripheral blood mononuclear cells (PBMCs) within 7 and 28 days in cell culture, and (2) flow cytometry analysis of peripheral blood CD34+ cells and of CD34+/KDR+/CD133+ cells.

**EPC culture assay**

Peripheral blood mononuclear cells will be isolated by density-gradient centrifugation with Ficoll-Paque Plus (Amersham Bioscience, Buckinghamshire, UK) from EDTA-anticoagulated blood (5 x 10^-2^ M final concentration). Immediately after isolation, PBMCs (4 x 10^6^) will be resuspended in 1mL of endothelial basal medium (EBM-2, Clonetics, Cambrex, East Rutherford, NJ) supplemented with EGM-2-MV-SingleQuots (Clonetics) and 10% FCS (Gibco, Life Technologies, Carlsbad, CA), and plated on 24-well culture dishes (TTP, Trasadingen, Switzerland) coated with human fibronectin (Sigma, St. Louis, MI). After three days of culture, non-adherent cells will be removed. On day seven, adherent cells will underwent cytochemical analysis after a thorough washing with PBS. To confirm the endothelial cell phenotype, cells will be incubated with 2.4 µg/mL 1,1'-dioctadecyl-3,3,3',3'- tetramethylindo-carbocyanine perchlorate-labeled acetylated low-density lipoprotein (Dil-Ac-LDL, Biomedical Technologies, Stoughton, MA) in fresh medium at 37°C for 3 hours. After fixation in 2% paraformaldehyde, cells will be counterstained with FITC-conjugated Ulex europaeus agglutinin (UEA)-1 lectin (10 µg/mL, Sigma) for 1 hour at room temperature (RT). Samples will be examined with an inverted fluorescent microscope and adherent cells staining positive for both Dil-Ac-LDL and lectin will be considered EPCs . The number of EPCs will be determined by counting three random high-power fields per subject by two independent investigators.

**Flow cytometry analysis**

A total of 2 x 10^6^ PBMCs will be incubated for 30 minutes in the dark at 4°C with saturating concentrations of the following monoclonal antibodies (mAb): PE-labeled anti-CD133 mAb (clone AC133, Miltenyi Biotec, Auburn, CA), APC-labeled anti-CD34 mAb (clone 581, Instrumentation Laboratories, Marseille, France), biotinylated anti-KDR mAb (clone KDR-2, Sigma) and corresponding isotype controls (mouse IgG1-biotinylated, clone MOPC21, mouse IgG1-PE, clone 679.1Mc7, mouse IgG1-APC, clone 679.1Mc7, all from Instrumentation Laboratories). After a single wash step with PBS, samples will be incubated for 15 minutes with PE-Cy5-conjugated streptavidin (Instrumentation Laboratories) at room temperature. Then, cells will be washed with PBS and fixed in 2% paraformaldehyde.

Quantitative analysis will be performed on a FACS-Calibur flow cytometer (Becton Dickinson, San Jose, CA) measuring 200,000 cells per sample. Data will be analyzed using Cellquest software (Becton Dickinson) by side scatter-fluorescence dot plot analysis. The number of EPCs will be defined as events triple-positive for CD34, CD133 and KDR with low cytoplasmatic granularity (low sideward scatter). CD34+ cells will be defined as hematopoietic stem cells.

**Graft function assessments**

To predict creatinine clearance and GFR the Cockcroft/Gault formula (corrected to a body surface area of 1.73m2) and the short MDRD prediction equation will be used at baseline, 3, 12, and 24 months post-study entry. Renal function will also be assessed by Cystatin C serum concentrations that will be measured at baseline, 3, 12, and 24 months post-study entry using a commercially available test kit.

**Humoral alloreactivity**

Alloreactivity against a panel of HLA antigens (panel reactive antibodies, PRA) will be assessed as a surrogate marker of humoral alloreactivity against the transplanted organ. PRA reactivity will be evaluated using flow cytometry-based PRA testing at baseline, and at month 3, 12, and 24.

**Panel reactive antibodies**

FlowPRA test: The FlowPRA screening test (One Lambda, Canoga Park, CA) will be performed according to the manufacturer's protocol. This test consists of a pool of 30 different microbead preparations coated with either purified HLA class I antigens or class II antigens from different cell lines covering all common HLA antigens. In brief, 9 µl serum or eluate are incubated with 0.5 µl FlowPRA class I (FL1-30) and 0.5 µl FlowPRA class II (FL2-30) beads. After incubation, beads are washed and then stained with appropriately pretitered FITC-conjugated F(ab´)2 goat anti-human IgG antibody. Fluorescence intensity is measured by flow cytometry using a FACSCalibur flow cytometer (Becton Dickinson, San Jose, CA). The major bead population will be gated on the forward- versus side-scatter dot plot. Then, two gates are set on the FL2 histogram to analyze class I (FL2-negative) and class II (FL2 high-fluorescent) beads separately. Alloantibody binding to class I or class II beads is evaluated according to FL1 fluorescence. The marker is set according to staining with a negative control serum.

**FlowPRAC4dtest**

For detection of complement product deposition, FlowPRA beads will be incubated with sera as described above. Beads are then washed three times and incubated with appropriately pretitered anti-C4d antibody. For control, beads are incubated with equal concentrations of rabbit IgG. After 30 min incubation at 4°C, beads are washed and then incubated with FITC-conjugated anti-rabbit IgG antibody for 30 min at 4°C. After washing, samples are analyzed by flow cytometry. As described for detection of alloantibody binding, class I and class II beads are distinguished on the FL2 histogram. The marker will be set according to the negative control, i.e. beads preincubated with negative control serum and incubated with non-binding rabbit IgG .

The degree of alloreactivity is defined by the percentage of PRA reactivity against HLA class I and HLA class II antigens (FlowPRA test: 0-100%; FlowPRA(C4d): 0-100%).

**Other assessments**

**Hypertension**

Hypertension is defined as a systolic blood pressure (SBP) of 140 mmHg or greater, diastolic blood pressure (DBP) of 90 mmHg or greater or taking anti-hypertensive medication. Hypertension will be classified according to the JNC VII Report Guidelines Treatment will be administered to maintain a target blood pressure less than approximately 130/80 mmHg. All medications for hypertension will be recorded.

**Hyperlipidemia**

Hyperlipidemia based on LDL, total cholesterol, and triglycerides will be defined according to the 2004 National Kidney Foundation guidelines for managment of hyperlipidemia in kidney transplant recipients

**Hyperglycemia and diabetes mellitus**

Diabetes mellitus will be classified according to the ADA criteria (Report of the expert committee on the diagnosis and classification of diabetes mellitus) .

### Table 1. Inclusion and exclusion criteria

### Inclusion Criteria

- Patient is recipient of a deceased or living donor renal transplant (including re-transplants)
- Patient was ≥18 years of age at the time of transplantation.
- Patient is at least 6 months post-transplant.
- Patient is on a cyclosporine-based immunosuppression regimen in combination with/without MMF and/or steroids at study entry.
- Patient has a functioning renal allograft and an estimated GFR ≥30 mL/min/1.73m^2^ within four weeks prior to study entry.
- Patient has a stable graft function without biopsy proven acute rejection episode within 3 months prior to study entry.
- Patient has not experienced a cardiovascular event (e.g. myocardial infarction, stroke, percutaneous angioplasty, bypass surgery,..) within 3 months prior to study entry.
- Patient has been fully informed and has given written informed consent according to ICH-GCP. Patient unable to write and/or read but who fully understands the oral information given by the investigator (or nominated representative) has given oral informed consent witnessed in writing by an independent person.
- Females are not pregnant and agree to practice effective birth control while receiving immunosuppressant medication.
- Patient has indications for conversion at the investigators discretion or is suffering from cyclosporine associated side effects like hypertension (≥130 and/or ≥80 mm Hg, with or without antihypertensive therapy), hyperlipidemia (LDL-cholesterol ≥ 100 mg/dl or triglycerides≥200 mg/dl and non-HDL-cholesterol ≥130 mg/dl) or cosmetic side effects.

### Exclusion Criteria

- Patient is recipient of a solid organ transplant other than the kidney.
- Patient has recurrence of primary renal disease, or de novo renal disease.
- Patient is pregnant or lactating.
- Patient had a known or suspected malignancy (except for treated squamous and basal cell skin cancers) <5 years before study entry or a history of post-transplant lymphoproliferative disease (PTLD).
- Patient has known hypersensitivity to tacrolimus, or any of the recipients of the drug.

**Table 2.** Indicators for safety

- Incidence of medical necessity to change immunosuppressive therapy
- Incidence of serious opportunistic infection
- Incidence of new-onset diabetes mellitus
- Incidence of hematologic adverse events including: thrombocytopenia, anemia, and neutropenia
- Incidence of cardiovascular events
- Incidence of malignancy, lymphoma and lymphoproliferative disease
- Incidence of gingival hyperplasia
- Incidence of hirsutism and alopecia
- Incidence of graft loss and death

# REFERENCES

1. Rosenzweig A. Endothelial progenitor cells. N Engl J Med 2003; 348: 581.

2. Asahara T, Murohara T, Sullivan A, Silver M, van der Zee R, Li T, et al. Isolation of putative progenitor endothelial cells for angiogenesis. Science 1997; 275: 964.

3. Peichev M, Naiyer AJ, Pereira D, Zhu Z, Lane WJ, Williams M, et al. Expression of VEGFR-2 and AC133 by circulating human CD34^+^ cells identifies a population of functional endothelial precursors. Blood 2000; 95: 952.

4. Urbich C, Dimmeler S. Endothelial progenitor cells. Characterization and role in vascular biology. Circ Res 2004; 95: 343.

5. Rehman J, Li J, Orschell CM, March KL. Peripheral blood "endothelial progenitor cells" are derived from monocyte/macrophages and secrete angiogenic growth factors. Circulation 2003; 107: 1164.

6. Rookmaaker MB, Vergeer M, van Zonneveld AJ, Rabelink TJ, Verhaar MC. Endothelial progenitor cells: mainly derived from the monocyte/macrophage-containing CD34^-^ mononuclear cell population and only in part from the hematopoietic stem cell-containing CD34+ mononuclear cell population. Circulation 2003; 108: e150; author reply e150.

7. Szmitko PE, Fedak PWM, Weisel RD, Stewart DJ, Kutryk MJB, Verma S. Endothelial progenitor cells: new hope for a broken heart. Circulation 2003; 107: 3093.

8. Tateishi-Yuyama E, Matsubara H, Murohara T, Ikeda U, Shintani S, Masaki H, et al. Therapeutic angiogenesis for patients with limb ischaemia by autologous transplantation of bone-marrow cells: a pilot study and a randomised controlled trial. Lancet 2002; 360: 427.

9. Assmus B, Schächinger V, Teupe C, Britten M, Lehmann R, Döbert N, et al. Transplantation of Progenitor Cells and Regeneration Enhancement in Acute Myocardial Infarction (TOPCARE-AMI). Circulation 2002; 106: 3009.

10. Strauer BE, Brehm M, Zeus T, Köstering M, Hernandez A, Sorg RV, et al. Repair of infarcted myocardium by autologous intracoronary mononuclear bone marrow cell transplantation in humans. Circulation 2002; 106: 1913.

11. Vasa M, Fichtlscherer S, Aicher A, Adler K, Urbich C, Martin H, et al. Number and migratory activity of circulating endothelial progenitor cells inversely correlate with risk factors for coronary artery disease. Circ Res 2001; 89: E1.

12. Hill JM, Zalos G, Halcox JPJ, Schenke WH, Waclawiw MA, Quyyumi AA, et al. Circulating endothelial progenitor cells, vascular function, and cardiovascular risk. N Engl J Med 2003; 348: 593.

13. Loomans CJM, de Koning EJ, Staal FJT, Rookmaaker MB, Verseyden C, de Boer HC, et al. Endothelial progenitor cell dysfunction: a novel concept in the pathogenesis of vascular complications of type 1 diabetes. Diabetes 2004; 53: 195.

14. Tepper OM, Galiano RD, Capla JM, Kalka C, Gagne PJ, Jacobowitz GR, et al. Human endothelial progenitor cells from type II diabetics exhibit impaired proliferation, adhesion, and incorporation into vascular structures. Circulation 2002; 106: 2781.

15. Schatteman GC, Hanlon HD, Jiao C, Dodds SG, Christy BA. Blood-derived angioblasts accelerate blood-flow restoration in diabetic mice. J Clin Invest 2000; 106: 571.

16. de Groot K, Bahlmann FH, Sowa J, Koenig J, Menne J, Haller H, et al. Uremia causes endothelial progenitor cell deficiency. Kidney Int 2004; 66: 641.

17. Vasa M, Fichtlscherer S, Adler K, Aicher A, Martin H, Zeiher AM, et al. Increase in circulating endothelial progenitor cells by statin therapy in patients with stable coronary artery disease. Circulation 2001; 103: 2885.

18. Heeschen C, Aicher A, Lehmann R, Fichtlscherer S, Vasa M, Urbich C, et al. Erythropoietin is a potent physiologic stimulus for endothelial progenitor cell mobilization. Blood 2003; 102: 1340.

19. Bahlmann FH, de Groot K, Duckert T, Niemczyk E, Bahlmann E, Boehm SM, et al. Endothelial progenitor cell proliferation and differentiation is regulated by erythropoietin. Kidney Int 2003; 64: 1648.

20. Bahlmann FH, de Groot K, Spandau JM, Landry AL, Hertel B, Duckert T, et al. Erythropoietin regulates endothelial progenitor cells. Blood 2004; 103: 921.

21. Khot UN, Khot MB, Bajzer CT, Sapp SK, Ohman EM, Brener SJ, et al. Prevalence of conventional risk factors in patients with coronary heart disease. JAMA 2003; 290: 898.

22. Kasiske BL, Chakkera HA, Roel J. Explained and unexplained ischemic heart disease risk after renal transplantation. J Am Soc Nephrol 2000; 11: 1735.

23. Zoccali C, Mallamaci F, Tripepi G. Novel cardiovascular risk factors in end-stage renal disease. J Am Soc Nephrol 2004; 15 (Suppl 1): S77.

24. Hackam DG, Anand SS. Emerging risk factors for atherosclerotic vascular disease: a critical review of the evidence. JAMA 2003; 290: 932.

25. Foley RN, Parfrey PS, Sarnak MJ. Epidemiology of cardiovascular disease in chronic renal disease. J Am Soc Nephrol 1998; 9 (Suppl 2): S16.

26. Krämer BK, Zülke C, Kammerl MC, Schmidt C, Hengstenberg C, Fischereder M, et al. Cardiovascular risk factors and estimated risk for CAD in a randomized trial comparing calcineurin inhibitors in renal transplantation. Am J Transplant 2003; 3: 982.

27. Artz MA, Boots JM, Ligtenberg G, Roodnat JI, Christiaans MHL, Vos PF, et al. Improved cardiovascular risk profile and renal function in renal transplant patients after randomized conversion from cyclosporine to tacrolimus. J Am Soc Nephrol 2003; 14: 1880.

28. Steiner S, Winkelmayer WC, Kleinert J, Grisar J, Seidinger D, Kopp CW, et al. Endothelial progenitor cells in kidney transplant recipients. Transplantation 2006; 81: 599.

29. Cockcroft DW, Gault MH. Prediction of creatinine clearance from serum creatinine. Nephron 1976; 16: 31.

30. Levey AS. Clinical practice. Nondiabetic kidney disease. N Engl J Med 2002; 347: 1505.

31. Wahrmann M, Exner M, Regele H, Derfler K, Kormoczi GF, Lhotta K, et al. Flow cytometry based detection of HLA alloantibody mediated classical complement activation. J Immunol Methods 2003; 275: 149.

32. Chobanian AV, Bakris GL, Black HR, Cushman WC, Green LA, Izzo JL, Jr., et al. The Seventh Report of the Joint National Committee on Prevention, Detection, Evaluation, and Treatment of High Blood Pressure: the JNC 7 report. JAMA 2003; 289: 2560.

33. NKF. Clinical practice guidelines for managing dyslipidemias in kidney transplant patients: a report from the managing dyslipidemias in chronic kidney disease work group of the national kidney foundation kidney disease outcomes quality initiative. Am J Transplant 2004; 4 (Suppl 7): 13.

34. American Diabetes Association: clinical practice recommendations 2002. Diabetes Care 2002; 25 Suppl 1: S1.
