# Supplementary material for: Late Conversion of Kidney Transplant Recipients from Ciclosporin to Tacrolimus Improves Graft Function: Results from a Randomized Controlled Trial
Source: PLoS One. 2015 Aug 13;10(8):e0135674. doi: 10.1371/journal.pone.0135674 (PMC4535983; doi:10.1371/journal.pone.0135674)
Supplement: S1 Table — Additional information about the two models from Table 2. (DOCX) [file pone.0135674.s004.docx]

S1 Table. Multivariable Data Analysis Results. Additional information about the two models from Table 2.

Model for eGFR_MDRD_

| **Covariance Parameter Estimates** | | | |
| --- | --- | --- | --- |
| **Cov Parm** | **Subject** | **Estimate** | **Standard Error** |
| **id(group)** |  | 21.1802 | 4.1080 |
| **SP(POW)** | **id(group)** | 0.6951 | 8.1712 |
| **Residual** |  | 35.2809 | 3.0085 |

| **Solutions for Fixed Effects** | | | | | | | |
| --- | --- | --- | --- | --- | --- | --- | --- |
| **Effect** | **group** | **time** | **Estimate** | **Standard Error** | **DF** | **t Value** | **Pr > \|t\|** |
| **Intercept** |  |  | 2.3448 | 1.7698 | 160.9 | 1.32 | 0.1871 |
| **group** | **0** |  | -3.9442 | 1.3706 | 324.3 | -2.88 | 0.0043 |
| **group** | **1** |  | 0 | . | . | . | . |
| **time** |  | **12** | 0.2718 | 0.8605 | 276.1 | 0.32 | 0.7523 |
| **time** |  | **52** | 0.7022 | 0.8623 | 275.1 | 0.81 | 0.4162 |
| **time** |  | **104** | 0 | . | . | . | . |
| **group*time** | **0** | **12** | 0.9085 | 1.5268 | 276.2 | 0.60 | 0.5523 |
| **group*time** | **0** | **52** | -1.0318 | 1.5284 | 275.9 | -0.68 | 0.5002 |
| **group*time** | **0** | **104** | 0 | . | . | . | . |
| **group*time** | **1** | **12** | 0 | . | . | . | . |
| **group*time** | **1** | **52** | 0 | . | . | . | . |
| **group*time** | **1** | **104** | 0 | . | . | . | . |
| **mdrd0** |  |  | 0.9968 | 0.03365 | 137.2 | 29.62 | <.0001 |

Model for eGFR_CKD-EPI_

| **Covariance Parameter Estimates** | | | |
| --- | --- | --- | --- |
| **Cov Parm** | **Subject** | **Estimate** | **Standard Error** |
| **id(group)** |  | 25.5558 | 4.9052 |
| **SP(POW)** | **id(group)** | 0.6948 | 8.2550 |
| **Residual** |  | 41.3987 | 3.5309 |

| **Solutions for Fixed Effects** | | | | | | | |
| --- | --- | --- | --- | --- | --- | --- | --- |
| **Effect** | **group** | **time** | **Estimate** | **Standard Error** | **DF** | **t Value** | **Pr > \|t\|** |
| **Intercept** |  |  | 3.1414 | 1.8775 | 162 | 1.67 | 0.0962 |
| **group** | **0** |  | -4.4747 | 1.4903 | 322.3 | -3.00 | 0.0029 |
| **group** | **1** |  | 0 | . | . | . | . |
| **time** |  | **12** | 0.3597 | 0.9321 | 276 | 0.39 | 0.6999 |
| **time** |  | **52** | 0.8311 | 0.9341 | 274.9 | 0.89 | 0.3744 |
| **time** |  | **104** | 0 | . | . | . | . |
| **group*time** | **0** | **12** | 0.9895 | 1.6539 | 276.1 | 0.60 | 0.5502 |
| **group*time** | **0** | **52** | -1.0281 | 1.6557 | 275.7 | -0.62 | 0.5352 |
| **group*time** | **0** | **104** | 0 | . | . | . | . |
| **group*time** | **1** | **12** | 0 | . | . | . | . |
| **group*time** | **1** | **52** | 0 | . | . | . | . |
| **group*time** | **1** | **104** | 0 | . | . | . | . |
| **epi0** |  |  | 0.9849 | 0.03314 | 137.2 | 29.72 | <.0001 |
